# Engineered Recombinant PON1-OPH Fusion Hybrids: Potentially Effective Catalytic Bioscavengers against Organophosphorus Nerve Agent Analogs

**DOI:** 10.4014/jmb.2006.06044

**Published:** 2020-10-30

**Authors:** Nari Lee, Hyeongseok Yun, Chan Lee, Yikjae Lee, Euna Kim, Sumi Kim, Hyoeun Jeon, Chiho Yu, Jaerang Rho

**Affiliations:** 1 Department of Microbiology and Molecular Biology, Chungnam National University, Daejeon 3434, Republic of Korea; 2 Agency for Defense Development, P.O. Box 35, Yuseong, Daejeon 34186, Republic of Korea

**Keywords:** Bioscavenger, paraoxonase 1, organophosphorus hydrolase, phosphotriesterase, organophosphorus compound, nerve agent

## Abstract

Organophosphorus nerve agents (OPNAs), including both G- and V-type nerve agents such as sarin, soman, tabun and VX, are extremely neurotoxic organophosphorus compounds. Catalytic bioscavengers capable of hydrolyzing OPNAs are under development because of the low protective effects and adverse side effects of chemical antidotes to OPNA poisoning. However, these bioscavengers have certain limitations for practical application, including low catalytic activity and narrow specificity. In this study, we generated a fusion-hybrid form of engineered recombinant human paraoxonase 1 (rePON1) and bacterial organophosphorus hydrolase (OPH), referred to as GV-hybrids, using a flexible linker to develop more promising catalytic bioscavengers against a broad range of OPNAs. These GV-hybrids were able to synergistically hydrolyze both G-type OPNA analogs (paraoxon: 1.7 ~ 193.7-fold, *p*-nitrophenyl diphenyl phosphate (PNPDPP): 2.3 ~ 33.0-fold and diisopropyl fluorophosphates (DFP): 1.4 ~ 22.8-fold) and V-type OPNA analogs (demeton-Smethyl (DSM): 1.9 ~ 34.6-fold and malathion: 1.1 ~ 4.2-fold above) better than their individual enzyme forms. Among the GV-hybrid clones, the GV7 clone showed remarkable improvements in the catalytic activity toward both G-type OPNA analogs (*k*_cat_/*K*_m_(10^6^M^-1^min^-1^): 59.8 ± 0.06 (paraoxon), 5.2 ± 0.02 (PNPDPP) and 47.0 ± 6.0 (DFP)) and V-type OPNA analogs (*k*_cat_/*K*_m_(M^-1^min^-1^): 504.3 ± 48.5 (DSM) and 1324.0 ± 47.5 (malathion)). In conclusion, we developed GV-hybrid forms of rePON1 and bacterial OPH mutants as effective and suitable catalytic bioscavengers to hydrolyze a broad range of OPNA analogs.

## Introduction

Organophosphorus compounds (OPs) are phosphorus-containing organic compounds such as parathion, malathion and chlorpyrifos that have been widely used as pesticides and insecticides [1]. Highly toxic OPs strongly inhibit acetylcholinesterase activity in the synapse, which leads to acetylcholine accumulation, thereby causing overstimulation of cholinergic receptors [1]. Thus, OP poisoning following skin exposure, inhalation, or ingestion causes severe cholinergic toxicity, resulting in muscle weakness and fatigue, salivation, convulsion, painful cramps, or even respiratory or cardiac muscle paralysis, leading to death [1]. Organophosphorus nerve agents (OPNAs) are the most neurotoxic OPs and are extremely toxic at very low doses. OPNAs, as chemical warfare agents, are considered one of the greatest health security threats globally [2, 3]. OPNAs are traditionally divided into two classes, G- and V-type agents, based on structural and functional similarities [1-3]. G-type OPNAs, such as sarin (GB), soman (GD) and tabun (GA), are volatile and less persistent in the environment, whereas V-type OPNAs, such as VX and VR, are less volatile and more stable: V-type agents are more toxic than G-type agents [1-3].

Anticholinergic drugs and oximes are commonly used to treat OPNA exposure, but these treatments are not sufficient to protect against OPNA poisoning and elicit many adverse side effects [3]. To overcome these limitations, the development of bioscavengers capable of hydrolyzing OPNAs has emerged as an alternative approach to protect against OPNA poisoning [2-4]. The G117H variant of human butyrylcholinesterase (BChE) has been developed as a catalytic bioscavenger with remarkably improved hydrolytic activity against OPs such as paraoxon and echothiophate [5]. The BChE-G117H or -G117H/E197Q variant can also hydrolyze OPNAs, including GB, GD and VX, but it is not sufficient for clinical use [5, 6]. Hence, several human enzymes, including paraoxonase 1 (PON1) and prolidase, and bacterial enzymes, including organophosphorus hydrolase (OPH, also called phosphotriesterase) and organophosphorus acid anhydrolase (OPAA), are under development as potential catalytic bioscavengers [2-4]. However, thus far, whether these catalytic bioscavenger candidates can acquire enhanced catalytic efficiency (*k*_cat_/*K*_m_value: >10^6^M^-1^min^-1^) with a broad spectrum (G- and V-type OPNAs) of substrate specificity through directed evolution studies remains a great concern.

Human PON1 is a major candidate catalytic bioscavenger currently in development to protect against OPNA poisoning [3, 4]. Human PON1 is primarily produced by the liver and is a high-density lipoprotein-associated hydrolytic enzyme in the serum with a wide range of substrates [7]. Human PON1 has ~240-fold greater catalytic activity toward paraoxon than the BChE-G117H mutant [7]. The purified PON1 derived from human plasma has shown protective effects with increased survival rate and reduced symptoms against exposure to OPNAs such as GB and GD in an experimental animal model [8]. However, the recombinant human PON1 (rePON1) expressed in *E. coli*is not soluble due to aggregation and a defect in glycosylation [9]. This aggregation problem of rePON1 has been solved by random DNA shuffling of human, mouse, rat and rabbit PON1: the rePON1 variant G3C9 (rePON1-G3C9 (gi: 40850544, ~85 % identical to the human PON1)) derived from random DNA shuffling is expressed in a soluble and functional form in *E. coli*[10]. Furthermore, rePON1-G3C9 is capable of hydrolyzing OPs or G-type OPNAs, including GB and GD, more efficiently than the human serum PON1 [10, 11]. Interestingly, it has been reported that the engineered mutant rePON1-4E9 or rePON1-IIG1, a derivative of rePON1-G3C9, exhibits highly improved hydrolytic activity (*k*_cat_/*K*_m_value: >10^7^M^-1^min^-1^) toward the coumarin analog of cyclosarin or G-type OPNAs [12, 13]. Although these engineered PON1 variants can hydrolyze G-type OPNAs with high catalytic efficiency, they have very low hydrolytic activities toward V-type OPNAs (rePON1-4E9, 8.0 × 10^1^M^-1^min^-1^; rePON1-IIG1, 1.43 × 10^2^M^-1^min^-1^) [12, 14]. This is one of the current limitations of using engineered rePON1 variants to develop broad-spectrum bioscavengers against OPNA poisoning [4].

Bacterial OPH isolated from *Pseudomonas diminuta*and *Flavobacterium*sp. has a broad range of substrate specificity for OPNA hydrolysis, whereas bacterial OPAA from *Alteromonas*sp. is not capable of hydrolyzing V-type OPNAs [15]. Bacterial OPH has ~140-fold greater catalytic activity toward VX than rePON1-G3C9 [11, 16]. Hence, bacterial OPH is considered a good candidate catalytic bioscavenger to detoxify V-type OPNAs, including VX and VR. In the last decade, much effort has been devoted to developing highly efficient bacterial OPH variants against V-type OPNA poisoning [4]. Interestingly, several engineered bacterial OPH variants that display highly improved catalytic activities, 150~600-fold greater V-type OPNA hydrolysis than their wild type, have been successfully developed [16-18]. Hence, to date, the engineered rePON1 and bacterial OPH variants are considered the most advanced catalytic bioscavengers to protect against OPNA poisoning [19]. The engineered rePON1 variants are suitable catalytic bioscavengers for detoxifying G-type OPNAs, while the engineered bacterial OPH variants are thus far the only catalytic bioscavengers that can efficiently hydrolyze V-type OPNAs [2-4].

In this study, we designed a fusion-hybrid form (hereafter referred to as GV-hybrids) of rePON1 and bacterial OPH mutants based on the catalytic characteristics of these advanced catalytic bioscavengers to develop a more promising catalytic bioscavenger against poisoning by a broad range of OPNAs. The C-terminal end of the engineered rePON1 mutants was fused with the N-terminal end of the engineered bacterial OPH mutants using a flexible linker to give a certain degree of flexibility or interaction between fused proteins. Here, we report that recombinant GV-hybrids exhibit improved catalytic activity against both G- and V-type OPNA analogs.

## Materials and Methods

### Chemicals

Paraoxon, diisopropyl fluorophosphate (DFP), malathion and demeton-S-methyl (DSM) were purchased from Sigma-Aldrich (USA). Other general chemicals were also obtained from Sigma-Aldrich. *p*-Nitrophenyl diphenyl phosphate (PNPDPP) was synthesized at the Agency for Defense Development, Korea, as previously described [20].

### Plasmids and Mutagenesis

For the wild-type (WT) rePON1 expression plasmid (rePON1-WT), the active region of human PON1 (amino acids 16-355) produced by PCR amplification using the pMTa-hygro-PON1-hFc plasmid reported in our previous study [21] was subcloned into the pET-21b vector (Novagen, USA) using Hind III and Sal I. The rePON1-4E9 clone (referred to as rePON1-G0 hereafter) reported by Gupta *et al*. [13] was generated by PCR-based site-directed mutagenesis as previously described [22]. For the development of engineered rePON1 mutants, random mutations were introduced into the rePON1-G0 clone by error-prone PCR-based mutagenesis as previously described [23]. Then, the randomly mutated rePON1 clones were subcloned into the pET-21b vector, and the mutated residues in rePON1 were identified by DNA sequence analysis (Solgent Co., Korea). The sequenced rePON1 mutant clones were transformed into the *E. coli*BL21 (DE3) strain. To obtain rePON1 mutants with improved paraoxon hydrolysis from the random mutagenesis, paraoxonase activities were analyzed using bacterial lysates harboring an individual clone of rePON1 mutants as previously described [12]. Briefly, BL21 (DE3) strains expressing the rePON1 mutant clone were harvested and lysed by bacterial lysis buffer (200 µl volume: 100 mM Tris-HCl (pH 8.0), 1 mM CaCl_2_, 0.2% Triton X-100 and 100 µg/ml lysozyme) with shaking at 300 rpm for 45 min at 37°C. The bacterial lysates (50 µl) prepared by centrifugation at 12,000 x g for 10 min at 4°C were used for the paraoxonase assay. For the WT bacterial OPH expression plasmid (OPH-WT), the active region of the OPH gene (amino acids 29-365) produced by PCR amplification from *Flavobacterium*sp. was subcloned into the pET-21b vector using Bam HI and Xho I. The expression plasmid of the bacterial OPH-L271/Y309A (or OPH-11M) clone (referred to as OPH-V0 hereafter) was generated by PCR-based site-directed mutagenesis as described in a previous study reported by Jeong *et al*. [16]. Since the hydrolysis of V-type OPNA analogs, such as DSM and malathion, was not detected by the rapid screening assay using bacterial lysates harboring an individual clone of OPH mutants, the target residues that may increase protein stability in OPH-V0 were selected based on in silico analysis to obtain improved OPH mutants for the hydrolysis of V-type OPNA analogs, as previously described [22]. The engineered bacterial OPH mutant clones were produced by PCR-based site-directed mutagenesis using the OPH-V0 clone as a template. For the construction of GV-hybrid expression plasmids, PCR-amplified rePON1 clones with a flexible linker (2×(G_4_S)) at the C-terminal end of rePON1 were fused with bacterial OPH mutant clones, and the fused GV-hybrids were finally subcloned into the pET-21b vector using Hind III and Xho I.

### Purification of Recombinant Proteins

The bacterial expression and purification of rePON1 was performed as previously described [22]. To improve the soluble expression of recombinant proteins, the expression plasmids of rePON1 mutants were transformed into *E. coli*BL21 (DE3) harboring groES, groEL and tig chaperones (Takara Bio, Japan). The rePON1 mutants were induced by 0.3 mM IPTG treatment at 30°C for 3 h. After IPTG induction, the cells were harvested by centrifugation and lysed by sonication. The rePON1 mutant proteins were purified using a Ni-NTA column (Qiagen, USA) according to the manufacturer’s instructions. The purified rePON1 mutant proteins were dialyzed in PON1 buffer (20 mM Tris-HCl (pH 8.0), 300 mM NaCl and 1 mM CaCl_2_) for 24 h at 4°C. For the bacterial expression and purification of bacterial OPH mutants, the expression plasmids of bacterial OPH mutants were transformed into *E. coli*BL21 (DE3) harboring groES and groEL chaperones (Takara Bio). The bacterial OPH mutant proteins were purified by Ni-NTA column and then dialyzed in OPH buffer (20 mM Tris-HCl (pH 8.0), 300 mM NaCl and 0.2 mM ZnCl_2_) for 24 h at 4°C. For the bacterial expression and purification of GV-hybrid mutants, the expression plasmids of GV-hybrid mutants were transformed into *E. coli*BL21 (DE3) harboring groES and groEL chaperones (Takara Bio). The purified proteins were dialyzed in GV buffer (20 mM Tris-HCl (pH 8.0), 300 mM NaCl, 1 mM CaCl_2_and 0.2 mM ZnCl_2_) for 24 h at 4°C. The purified proteins were analyzed by Coomassie Blue staining on 10% SDS-PAGE gels, and protein concentrations were measured using a Protein Assay Kit (Bio-Rad, USA) according to the manufacturer’s instructions.

### Enzymatic Activity

The catalytic efficiency of recombinant mutant proteins toward OPNA analogs was measured as previously described [16, 21, 22]. For the hydrolysis of paraoxon, the purified protein (0.1 µg) was incubated with paraoxon (0.02-0.8 mM) in a total volume of 200 µl of reaction buffer (50 mM Tris-HCl (pH 7.4), 10 mM CaCl_2_and 0.2 mM ZnCl_2_) at 25°C for 5 min. The enzymatic activity of paraoxon hydrolysis was measured at 412 nm, and the molar extinction coefficient of *p*-nitrophenol was 17,100 M^-1^cm^-1^. For the hydrolysis of DFP, the purified protein (0.5 µg) was incubated with DFP (0.05-1 mM) in a total volume of 200 µl of reaction buffer (0.004% phenol red, 2.0 mM HEPES (pH 8.0), 10 mM CaCl_2_and 0.2 mM ZnCl_2_) at 25°C for 5 min. The absorbance was measured spectrophotometrically at 422 nm, and the enzyme activity was calculated using the following equation: (ΔA_422_/Δt)/(volume of sample in µl×1.9×10^3^). For the hydrolysis of PNPDPP, the purified protein (0.3 µg) was incubated with 0.005-0.1 mM PNPDPP in 200 µl of reaction buffer (50 mM Tris-HCl (pH 7.4), 1% DMSO, 10 mM CaCl_2_and 0.2 mM ZnCl_2_) at 25°C for 5 min. The release of *p*-nitrophenol was measured spectrophotometrically at 412 nm. For the hydrolysis of DSM or malathion, the purified protein (5.0 µg) was incubated with DSM (0.6-3.6 mM) or malathion (0.2-2.4 mM) in a total volume of 200 µl of reaction buffer (50 mM Tris-HCl (pH 7.0), 100 mM NaCl, 0.5 mM 5´,5´-dithiobis(2-nitrobenzoic acid), 10 mM CaCl_2_and 0.2 mM ZnCl_2_) at 25°C for 10 min. The release of 2-nitro-5-thiobenzoate was monitored at 412 nm, and the molar extinction coefficient of 2-nitro-5-thiobenzonate was 13,600 M^-1^cm^-1^. The *k*_cat_and *K*_m_values were obtained using Michaelis-Menten steady state kinetics.

### Statistical Analysis

The presented data represent the means ± standard deviation (*n*= 3 per group). All of the experiments were performed at least three times. The Student’s *t*-test was used to determine the significance.

## Results

### Generation of Engineered rePON1 Mutants

The engineered clone rePON1-4E9 (hereafter referred to as rePON1-G0), which exhibits highly improved hydrolytic activity toward the coumarin analog of cyclosarin, was developed by Gupta *et al*. [13]. To further improve the catalytic activity of rePON1-G0 toward G-type OPNA analogs, we performed error-prone PCR-based mutagenesis to introduce a low range of random mutations (2-4 mutations/kb) in the coding region of rePON1-G0. The average rate of randomly introduced mutations in the rePON1-G0 was 2.1 mutations per gene. The mutated rePON1 proteins were expressed in *E. coli*BL21 (DE3), and paraoxonase activities in bacterial lysates were measured and compared to the activity in the bacterial lysate of the rePON1-G0 clone. From the results of screening 1,431 rePON1 mutant clones, we selected 12 clones, referred to as G1 to G12, with hydrolytic activities at least 1.5-fold higher than that of the rePON1-G0 clone. The mutated residues of the rePON1 mutants selected are shown in Table 1.

**Fig. 1:**
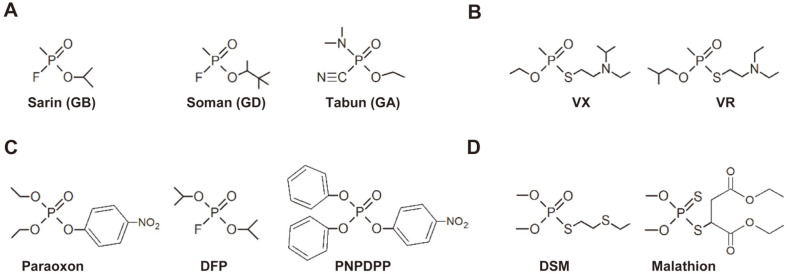
Chemical structures of organophosphorus nerve agents and their analogs. (**A**) Chemical structure of G-type nerve agents. (**B**) Chemical structure of V-type nerve agents. (**C**) Chemical structure of G-type nerve agent analogs. DFP, diisopropyl fluorophosphate. PNPDPP, p-nitrophenyl diphenyl phosphate. (**D**) Chemical structure of V-type nerve agent analogs. DSM, demeton-S-methyl.

To further examine the catalytic activities of selected rePON1 mutants toward G-type OPNA analogs, including paraoxon, PNPDPP and DFP (Fig. 1), we purified rePON1 mutant proteins from the bacterial lysates using Ni-NTA columns. To examine the catalytic efficiency of rePON1-G0 toward paraoxon hydrolysis, we first compared the *k*_cat_/*K*_m_values of the purified rePON1-G0 with those of the wild type (rePON1-WT). The *k*_cat_/*K*_m_values of rePON1-G0 (0.12 ± 0.01 × 10^6^M^-1^min^-1^) were 42.9-fold higher than those of rePON1-WT (0.003 ± 0.0001 × 10^6^M^-1^min^-1^). Then, we measured the paraoxon hydrolytic activity of the purified rePON1 mutant clones. In the mutant analyses for paraoxon hydrolysis, we observed that almost all the clones of rePON1 mutants, except for rePON1-G2 with low catalytic activity, had over 1.5-fold higher catalytic activity than rePON1-G0 (Table 1). Among these improved mutant clones, rePON1-G6, rePON1-G8, rePON1-G9, rePON1-G10 and rePON1-G11 had much higher catalytic activity for paraoxon hydrolysis (>3.0-fold higher, *k*_cat_/*K*_m_value: >0.36 ± 0.06 × 10^6^M^-1^min^-1^) than rePON1-G0 (Table 1). Moreover, compared to rePON1-WT, these improved mutant clones showed over 128.6-fold higher catalytic activity for paraoxon hydrolysis (Table 1). In thermal and pH stability assays, we observed that mutants rePON1-G6, rePON1-G8, rePON1-G9, rePON1-G10, and rePON1-G11 were relatively more stable than rePON1-G0 at 25-55°C, whereas these mutants had no changes in their catalytic activity compared to rePON1-G0 in a pH range of 6.0-9.0 (Fig. S1A). For the hydrolysis of PNPDPP, rePON1-G0 acquired considerable catalytic activity (0.23 ± 0.01 × 10^6^M^-1^min^-1^) compared to rePON1-WT, which had no such catalytic activity (Table 1). In the mutation analyses for PNPDPP hydrolysis, similar to the hydrolysis of paraoxon, we observed much higher catalytic activities (> 4.4-fold higher, *k*_cat_/*K*_m_value: > 0.98 ± 0.01 × 10^6^M^-1^min^-1^) in rePON1-G9, rePON1-G10, and rePON1-G11 than in rePON1-G0 (Table 1). These results indicate that the mutations S193A/H251Q/T257A/F264L in rePON1-G9, rePON1-G10, and rePON1-G11 are required for the improved catalytic efficiency of rePON1 toward paraoxon and PNPDPP hydrolysis. For the hydrolysis of DFP, the *k*_cat_/*K*_m_values of rePON1-G0 (1.40 ± 0.09 × 10^6^M^-1^min^-1^) were 5.9-fold higher than those of rePON1-WT (0.24 ± 0.01 × 10^6^M^-1^min^-1^). In the mutation analyses, we observed that the catalytic activity for DFP hydrolysis was slightly improved (1.37-fold higher, *k*_cat_/*K*_m_value: 1.92 ± 0.2 × 10^6^M^-1^min^-1^) only in the rePON1-G9 mutant compared to rePON1-G0, whereas the other rePON1 mutants showed slight decreases or no changes in their catalytic activity (Table 1). Thus, these results indicate that the catalytic activity of DFP hydrolysis was not greatly improved in the rePON1 mutants, except for rePON1-G9.

We next examined the catalytic activity of rePON1-G0 for the hydrolysis of V-type OPNA analogs, such as DSM and malathion (Fig. 1). The *k*_cat_/*K*_m_value of rePON1-G0 for DSM hydrolysis was significantly improved (23.3 ± 2.99 × M^-1^min^-1^) over that of rePON1-WT, which showed no catalytic activity (Table 1). In the mutation analyses, however, hydrolytic activity toward DSM was not detected in any of the rePON1 mutants tested, and only the rePON1-G12 clone maintained hydrolytic activity comparable to that of rePON1-G0 (Table 1). In addition, all rePON1 mutant clones tested had no hydrolytic activity toward malathion (Table 1). These results indicate that mutations in the rePON1 mutant clones negatively affect the catalytic activity for the hydrolysis of V-type OPNA analogs.

### Generation of Engineered Bacterial OPH Mutants

The engineered OPH-L271/Y309A (OPH-11M) clone (hereafter referred to as OPH-V0) that exhibits improved catalytic activity toward VX hydrolysis was developed by Jeong *et al*. [16]. To further improve the catalytic activity of OPH-V0 toward V-type OPNA analogs, the target residues that may increase protein stability in OPH-V0 were selected based on in silico analysis, as previously described [22]. We generated 6 mutant clones, referred to as OPH-V1 to OPH-V6, using PCR-based site-directed mutagenesis (Table 2). The mutated residues of the generated OPH mutant clones are shown in Table 2. We first compared the *k*_cat_/*K*_m_value of the purified OPH-V0 for DSM hydrolysis with that of the wild type (OPH-WT). The *k*_cat_/*K*_m_value of OPH-V0 for DSM hydrolysis was 83.5 ± 2.0 × M^-1^min^-1^, while the hydrolytic activity of OPH-WT was not detected (Table 2). To examine the catalytic activities of the OPH mutant clones toward V-type OPNA analogs, we next measured the DSM hydrolysis efficiency of the OPH mutant clones. OPH-V1, OPH-V2 and OPH-V3 had almost 2-fold to 2.84-fold higher catalytic activities for DSM hydrolysis than OPH-V0 (Table 2). Contrary to our expectation, however, the OPH-V4, OPH-V5 and OPH-V6 mutants showed no catalytic activity or no significant increase in the catalytic activity for DSM hydrolysis (Table 2). We obtained similar results in a malathion hydrolysis assay with OPH mutant clones (Table 2). Furthermore, in thermal and pH stability assays, we observed that OPH-V1, OPH-V2 and OPH-V3 mutants were relatively more stable than OPH-V0 at 35-60°C or in a pH range of 6.0-9.0 (Fig. S1B). These results indicate that mutations K77A/K159E/T173N/T177A in the OPH-V1, OPH-V2 and OPH-V3 clones are required for improved catalytic efficiency for the hydrolysis of V-type OPNA analogs.

We next examined the hydrolytic activity of OPH-V0 toward G-type OPNA analogs, including paraoxon, PNPDPP and DFP. The *k*_cat_/*K*_m_values of OPH-V0 toward G-type OPNA analogs were slightly decreased (0.26 ~ 0.93-fold) compared to those of OPH-WT (Table 2). To further examine the catalytic activities of OPH mutant clones toward G-type OPNA analogs, we measured the hydrolytic activity of the purified OPH mutant clones. The catalytic activities of OPH-V1, OPH-V2 and OPH-V3 clones for paraoxon hydrolysis were slightly increased (1.49 ~ 1.77-fold) compared to that of OPH-V0, while OPH-V4, OPH-V5 and OPH-V6 had decreased hydrolytic activities (Table 2). However, compared to the OPH-WT, the overall catalytic activities of all mutant clones for paraoxon hydrolysis were significantly decreased (Table 2). Similarly, we observed that the DFP hydrolytic activities of OPH-V1, OPH-V2 and OPH-V3 were slightly increased compared to those of OPH-V0 and OPH-WT (1.49 ~ 2.25-fold and 1.39 ~ 2.09-fold, respectively), while OPH-V4, OPH-V5 and OPH-V6 had decreased hydrolytic activities (Table 2). For the hydrolysis of PNPDPP, all of the OPH mutant clones had slightly increased hydrolytic activities (2.22 ~ 4.02-fold and 1.36 ~ 2.46-fold, respectively) compared to OPH-V0 or OPH-WT (Table 2). In summary, these results indicate that the engineered mutations in the OPH-V1, OPH-V2 and OPH-V3 mutant clones, which exhibit slightly improved catalytic activities toward G-type OPNA analogs compared to those of OPH-V0, may affect the catalytic efficiency or substrate binding specificity for the hydrolysis of G-type OPNA analogs.

### Generation of GV-Hybrids

**Fig. 2:**
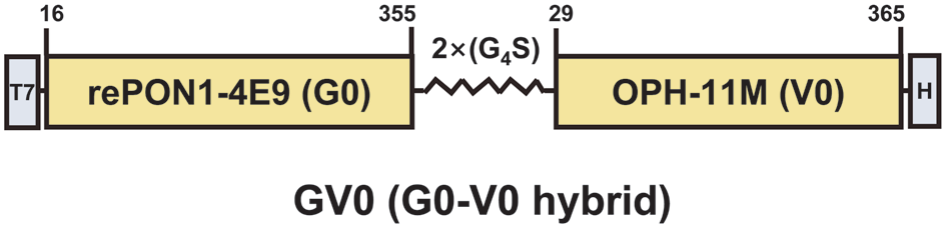
Schematic diagram of GV-hybrid construction. The numbers of amino acid residues in human rePON1 and bacterial OPH are shown at the top. T7, T7 tag epitope. H, 6×His epitope. 2× (G_4_S), a flexible linker.

The development of highly effective and broad-spectrum bioscavengers is necessary for efficient protection against OPNA poisoning [2, 4]. To address these issues, we generated GV-hybrids by the fusion of rePON1 mutants with bacterial OPH mutants using the flexible linker (2×(G_4_S)) at the C-terminal end of rePON1 (Fig. 2). As a preliminary experiment, we analyzed the impact of the G_4_S-linker length and the order of recombinant enzymes in GV-hybrid fusion protein construction. GV-hybrid clones with 2×(G_4_S)-linker had much higher catalytic activity for paraoxon or malathion hydrolysis than 1×(G_4_S) linker or 3×(G_4_S) linker clones (Table S1). In addition, GV-hybrids also exhibited much higher catalytic activity than VG-hybrids (OPH-(2×(G_4_S))-rePON1), which are in reverse order of the designed GV-hybrids (Table S2).

To compare the catalytic efficiency of the purified GV-hybrids, we first analyzed the *k*_cat_/*K*_m_values of GV0, a fusion of rePON1-G0 and OPH-V0, toward G-type OPNA analogs. For the hydrolysis of paraoxon and PNPDPP, the *k*_cat_/*K*_m_values of GV0 were 17.2- or 33.0-fold higher than those of rePON1-G0 (Table 3). However, the DFP hydrolytic activity of GV0 was slightly decreased (0.6-fold) compared to that of rePON1-G0 (Table 3). We next examined the catalytic activity of the GV-hybrids for paraoxon hydrolysis. All GV-hybrid clones had significantly higher catalytic activity (2.0 ~ 11.3-fold) for paraoxon hydrolysis than GV0 (Table 3). Among the GV-hybrid clones, the GV7 clone showed the highest catalytic activity (59.8 ± 0.06 × 10^6^M^-1^min^-1^) for paraoxon hydrolysis. The GV7 clone had 193.7- or 18.7-fold higher catalytic activity than rePON1-G0 or OPH-V0, respectively, for paraoxon hydrolysis (Table 3). For the hydrolysis of DFP, similar to paraoxon hydrolysis, all GV-hybrid clones also had significantly higher catalytic activity (4.2 ~ 40.6-fold) than GV0 (Table 3). However, the hydrolytic activities of GV-hybrids toward PNPDPP were slightly decreased (0.4 ~ 0.9-fold) compared to that of GV0 (Table 3). These results indicate that GV-hybrid clones had significantly improved catalytic efficiency for paraoxon and DFP hydrolysis compared to rePON1-G0 or GV0, but not for PNPDPP hydrolysis.

We next examined the hydrolytic activities of GV-hybrids toward the V-type OPNA analogs. For the hydrolysis of DSM, the *k*_cat_/*K*_m_values of the GV0 were decreased (0.3- or 0.1-fold, respectively) compared to those of rePON1-G0 or OPH-V0 (Table 3). However, almost all GV-hybrids had significantly higher catalytic activity than did rePON1-G0 or OPH-V0 (14.2 ~ 34.6-fold or 2.7 ~ 6.5-fold, respectively) for DSM hydrolysis (Table 3). Among the GV-hybrids, the GV7 clone showed the highest catalytic activity (504.3 ± 48.5 × M^-1^min^-1^) for DSM hydrolysis (Table 3). We obtained similar results in a malathion hydrolysis assay with GV-hybrid clones (Table 3). These results indicate that the GV-hybrid clones had significantly improved catalytic efficiency for the hydrolysis of V-type OPNA analogs compared to the hydrolytic activity of OPH-V0 or GV0.

## Discussion

The engineered variants of human PON1 capable of hydrolyzing G-type OPNAs are good candidate catalytic bioscavengers currently in development to protect against OPNA poisoning [3, 4]. In this study, we generated twelve rePON1 mutant clones (rePON1-G1 to rePON1-G12) to improve the catalytic activity of rePON1 for the hydrolysis of G-type OPNA analogs. In the rePON1 mutation analyses, we observed that the mutations in rePON1-G9, rePON1-G10 or rePON1-G11 are clearly involved in the improved catalytic efficiency for the hydrolysis of G-type OPNA analogs (Table 1). Thus, it is possible to postulate that the mutations, including S193A, H251Q, T257A and F264L, are closely associated with the substrate specificity/binding for the hydrolysis of G-type OPNA analogs. However, hydrolytic activity for DSM or malathion hydrolysis was not detected in these rePON1 mutants (Table 1). Interestingly, rePON1-G9, rePON1-G10, and rePON1-G11 acquired a common mutation, namely, S193A. Otto *et al*. also reported [24] that human PON1 with the S193A/G mutation has significantly decreased catalytic activity for VX and VR hydrolysis. Thus, the S193A substitution in rePON1 mutants may negatively affect the substrate specificity/binding for the hydrolysis of V-type OPNA analogs.

The engineered variants of bacterial OPH are more suitable candidate catalytic bioscavengers for detoxifying V-type OPNAs, including VX and VR [2-4]. In this study, we developed six OPH mutant clones (OPH-V1 to OPH-V6) to improve the catalytic activity of OPH-V0 for the hydrolysis of V-type OPNA analogs. Among the OPH mutant clones, OPH-V1, OPH-V2, and OPH-V3 showed improved catalytic efficiency for DSM or malathion hydrolysis compared to OPH-V0, while no hydrolytic activity or no enhanced hydrolytic activity compared to OPH-V0 was detected for OPH-V4, OPH-V5, and OPH-V6 (Table 2). OPH-V4, OPH-V5 and OPH-V6 have acquired a common mutation, namely, H254G. H254 is a key residue in the active site of OPH-WT that participates in proton shuttling from the active site in the hydrolysis of organophosphate triesters [25]. Interestingly, Bigley *et al*. reported [17, 18] that the OPH-QF variant with H254Q cannot protonate the leaving group in the active site, while F132V substitution significantly improved the catalytic activity for VX hydrolysis. Moreover, it was recently proposed by Goldsmith *et al*. [26] that the amino acid occupying the key position 254 seems to dictate the occupancy at position 132 in the active site of OPH variants. The F132E substitution is only observed in OPH variants with H254G or H254D mutations, while the OPH variants with H254Q also have F132D [26, 27]. Consequently, the F132D mutation is negatively involved in the VX hydrolysis [26, 27]. In our current study, therefore, we expect that the combination of F132D and H254D in OPH-V4, OPH-V5 and OPH-V6 may also have deleterious effects on DSM or malathion hydrolysis.

Many studies were conducted in the last decade to develop efficient catalytic bioscavengers capable of preventing OPNA poisoning [2-4]. In these research efforts, several catalytic bioscavengers, including human BChE, PON1, and bacterial OPH variants that are more suitable for enhancing OPNA hydrolysis, have been developed. Considering the extreme toxicity and relative structural diversity of OPNAs, nevertheless, there are still several limitations, including low catalytic activity and narrow specificity, on the use of the currently developed catalytic bioscavengers against OPNA poisoning [2, 4]. To overcome these limitations, we introduced a fusion-hybrid strategy with directed evolution in an enzyme engineering approach to improve catalytic activity and broad substrate specificity. In our current study, we developed bifunctional chimeric enzymes called GV-hybrids, which hydrolyze both G- and V-type OPNA analogs more efficiently. The fusion-hybrid form is generated by ‘end-to-end’ fusion in which the C-terminal end of a rePON1 mutant is linked to the N-terminal residue of the bacterial OPH mutants using a flexible linker to permit a certain degree of flexibility, mobility or interaction between fused enzymes. Interestingly, we observed synergistic effects in the hydrolysis of both G- and V-type OPNA analogs by almost all GV-hybrid clones compared to the individual enzyme form of rePON-G0 or OPH-V0 alone (Table 3). In particular, the GV7-hybrid clone showed remarkable improvements in catalytic activities toward both G- and V-type OPNA analogs (Tables 3). Thus, based on these observations, it is possible to presume that a conformational change in fusion-hybrid forms by the synergistic combination of rePON1 and bacterial OPH mutants may contribute to obtaining the acquired catalytic activity of the GV-hybrid with further improved hydrolysis toward both G- and V-type OPNA analogs. In our current study, however, it is not yet clear how the catalytic activities of GV-hybrids are synergistically improved by the generation of a fusion-hybrid form. Thus, further studies are required to elucidate the structural basis by which the fusion-hybrid form can synergistically enhance the hydrolytic activity toward OPNA analogs to understand conformational effects, including specificity, stability and substrate binding.

In conclusion, we have developed a fusion-hybrid form (GV-hybrids) by traditional end-to-end fusion of the rePON1 and bacterial OPH mutants using a flexible linker. The bifunctional chimeric GV-hybrid enzymes exhibited increased catalytic efficiency for the hydrolysis of both G- and V-type OPNA analogs. The results seem to indicate that GV-hybrids are potentially more effective and more suitable catalytic bioscavengers than other candidates to prevent poisoning by a broad range of OPNAs.

## Supplemental Material

Supplementary data for this paper are available on-line only at http://jmb.or.kr.

## Figures and Tables

**Table 1:** Catalytic rates of rePON1 variants.

Substrate	Clone	Mutational composition^a^	*k*_cat_(min^-1^)	*K*_m_(μM)	*k*_cat_/*K*_m_ (×10^6^M^-1^min^-1^)	Fold increase to WT	Fold increase to G0

Paraoxon	WT		4.7 ± 0.13	1682.0 ± 96.2	0.003 ± 0.0001	1.0	-
	G0	(rePON1-4E9)	6.3 ± 0.09	52.4 ± 1.5	0.12 ± 0.01	42.9	1.0
	G1	H251Q	7.6 ± 0.14	40.3 ± 0.7	0.19 ± 0.01	67.9	1.6
	G2	N43K/Q329H	2.6 ± 0.12	39.6 ± 6.0	0.07 ± 0.01	25.0	0.6
	G3	S193A	10.6 ± 0.09	54.8 ± 2.3	0.19 ± 0.01	67.9	1.6
	G4	F264L	8.3 ± 0.06	25.6 ± 0.7	0.32 ± 0.01	114.3	2.7
	G5	T257A	8.6 ± 0.32	39.9 ± 3.1	0.22 ± 0.01	78.6	1.8
	G6	S193A/F264L	8.0 ± 0.25	18.3 ± 5.5	0.43 ± 0.12	153.6	3.6
	G7	S193A/H251Q	9.8 ± 0.60	32.9 ± 0.2	0.30 ± 0.02	107.1	2.5
	G8	H251Q/F264L	7.6 ± 0.36	21.3 ± 4.7	0.36 ± 0.06	128.6	3.0
	G9	S193A/H251Q/F264L	19.3 ± 0.19	33.6 ± 0.2	0.57 ± 0.01	203.6	4.8
	G10	S193A/T257A/F264L	19.6 ± 0.79	37.0 ± 5.0	0.53 ± 0.05	189.3	4.4
	G11	S193A/H251Q/T257A	16.1 ± 0.35	40.4 ± 6.3	0.40 ± 0.07	142.8	3.3
	G12	H251Q/T257A/F264L	21.8 ± 0.22	67.7 ± 8.9	0.32 ± 0.04	114.3	2.7
PNPDPP	WT		N.D.	N.D.	N.D.	-	-
	G0	(rePON1-4E9)	4.2 ± 0.12	18.9 ± 1.8	0.23 ± 0.01	-	1.0
	G1	H251Q	3.4 ± 0.11	12.9 ± 1.0	0.26 ± 0.01	-	1.2
	G2	N43K/Q329H	N.D.	N.D.	N.D.	-	-
	G3	S193A	6.8 ± 0.06	18.6 ± 0.6	0.37 ± 0.02	-	1.6
	G4	F264L	5.4 ± 0.08	12.1 ± 0.3	0.45 ± 0.02	-	2.0
	G5	T257A	5.0 ± 0.01	15.2 ± 1.7	0.33 ± 0.04	-	1.5
	G6	S193A/F264L	3.3 ± 0.10	7.9 ± 0.1	0.41 ± 0.01	-	1.8
	G7	S193A/H251Q	6.0 ± 0.19	16.3 ± 0.9	0.37 ± 0.01	-	1.6
	G8	H251Q/F264L	4.7 ± 0.01	10.8 ± 0.1	0.44 ± 0.01	-	1.9
	G9	S193A/H251Q/F264L	12.9 ± 0.26	9.9 ± 0.6	1.31 ± 0.11	-	5.8
	G10	S193A/T257A/F264L	15.3 ± 0.02	11.8 ± 0.2	1.30 ± 0.03	-	5.8
	G11	S193A/H251Q/T257A	12.4 ± 0.13	12.7 ± 0.1	0.98 ± 0.01	-	4.4
	G12	H251Q/T257A/F264L	10.1 ± 0.81	17.8 ± 2.6	0.57 ± 0.04	-	2.5
DFP	WT		406.5 ± 16.7	1717.0 ± 25.5	0.24 ± 0.01	1.00	-
	G0	(rePON1-4E9)	155.3 ± 6.1	111.1 ± 11.2	1.40 ± 0.09	5.93	1.00
	G1	H251Q	144.6 ± 0.3	126.0 ± 60.5	1.30 ± 0.62	5.48	0.92
	G2	N43K/Q329H	N.D.	N.D.	N.D.	-	-
	G3	S193A	180.0 ± 21.2	131.3 ± 31.5	1.39 ± 0.17	5.88	0.99
	G4	F264L	195.6 ± 9.8	158.4 ± 21.1	1.25 ± 0.23	5.28	0.89
	G5	T257A	242.9 ± 24.5	281.4 ± 23.5	0.86 ± 0.01	3.64	0.62
	G6	S193A/F264L	N.D.	N.D.	N.D.	-	-
	G7	S193A/H251Q	163.8 ± 31.1	370.9 ± 24.4	0.53 ± 0.26	2.23	0.38
	G8	H251Q/F264L	132.9 ± 15.0	170.5 ± 18.1	0.79 ± 0.17	3.33	0.56
	G9	S193A/H251Q/F264L	347.3 ± 12.0	181.2 ± 12.2	1.92 ± 0.20	8.13	1.37
	G10	S193A/T257A/F264L	238.7 ± 28.4	169.9 ± 46.2	1.44 ± 0.22	6.07	1.02
	G11	S193A/H251Q/T257A	289.1 ± 10.4	212.2 ± 33.5	1.38 ± 0.17	5.81	0.98
	G12	H251Q/T257A/F264L	299.5 ± 8.7	304.4 ± 14.8	0.98 ± 0.02	4.16	0.70

Substrate	Clone	Mutational composition^a^	*k*_cat_(min^-1^)	*K*_m_(μM)	*k*_cat_/*K*_m_ (M^-1^min^-1^)	Fold increase to WT	Fold increase to G0

DSM	WT		N.D.	N.D.	N.D.	-	-
	G0	(rePON1-4E9)	0.027 ± 0.003	1161 ± 272	23.3 ± 2.99	-	1.00
	G9	S193A/H251Q/F264L	N.D.	N.D.	N.D.	-	-
	G10	S193A/T257A/F264L	N.D.	N.D.	N.D.	-	-
	G11	S193A/H251Q/T257A	N.D.	N.D.	N.D.	-	-
	G12	H251Q/T257A/F264L	0.038 ± 0.004	1700 ± 412	22.6 ± 2.85	-	0.97
Malathion	WT		N.D.	N.D.	N.D.	-	-
	G0	(rePON1-4E9)	N.D.	N.D.	N.D.	-	-
	G9	S193A/H251Q/F264L	N.D.	N.D.	N.D.	-	-
	G10	S193A/T257A/F264L	N.D.	N.D.	N.D.	-	-
	G11	S193A/H251Q/T257A	N.D.	N.D.	N.D.	-	-
	G12	H251Q/T257A/F264L	N.D.	N.D.	N.D.	-	-

^a^Substitutions relative to G0 (rePON1-4E9). WT, wild type; N.D., not determined.

**Table 2:** Catalytic rates of OPH variants.

Substrate	Clone	Mutational composition^a^	*k*_cat_(min^-1^)	*K*_m_(μM)	*k*_cat_/*K*_m_ (M^-1^min^-1^)	Fold increase to WT	Fold increase to V0

DSM	WT		N.D.	N.D.	N.D.	-	-
	V0	(OPH-11M)	0.13 ± 0.01	1513.5 ± 108.2	83.5 ± 2.0	-	1.00
	V1	K159E/T177A	0.49 ± 0.05	2946.0 ± 227.7	165.9 ± 2.6	-	1.99
	V2	K77A/K159E/T177A	0.37 ± 0.01	1580.5 ± 303.3	237.3 ± 37.3	-	2.84
	V3	K159E/T173N/T177A	0.29 ± 0.02	1508.0 ± 128.7	193.3 ± 6.3	-	2.32
	V4	K159E/T177A/H254G	0.69 ± 0.08	7425.5 ± 245.2	93.2 ± 4.2	-	1.12
	V5	K159E/T173N/T177A/H254G	N.D.	N.D.	N.D.	-	N.D.
	V6	K77A/K159E/T173N/T177A/H254G	N.D.	N.D.	N.D.	-	N.D.
Malathion	WT		N.D.	N.D.	N.D.	-	-
	V0	(OPH-11M)	0.55 ± 0.01	1709.5 ± 316.4	328.8 ± 52.6	-	1.00
	V1	K159E/T177A	2.25 ± 0.50	2698.5 ± 836.5	845.7 ± 75.3	-	2.57
	V2	K77A/K159E/T177A	2.42 ± 0.25	3459.5 ± 511.2	700.5 ± 31.7	-	2.13
	V3	K159E/T173N/T177A	3.09 ± 0.09	3329.0 ± 159.8	928.8 ± 18.0	-	2.82
	V4	K159E/T177A/H254G	1.64 ± 0.07	5188.0 ± 411.2	374.4 ± 14.6	-	1.14
	V5	K159E/T173N/T177A/H254G	0.71 ± 0.02	2240.0 ± 103.6	329.4 ± 6.00	-	1.00
	V6	K77A/K159E/T173N/T177A/H254G	0.78 ± 0.06	2863.5 ± 492.9	275.5 ± 26.8	-	0.84

Substrate	Clone	Mutational composition^a^	*k*_cat_(min^-1^)	*K*_m_(μM)	*k*_cat_/*K*_m_ (×10^6^M^-1^min^-1^)	Fold increase to WT	Fold increase to V0

Paraoxon	WT		2353.2 ± 92.6	204.7 ± 17.0	11.5 ± 0.5	1.00	-
	V0	(OPH-11M)	752.1 ± 42.3	250.8 ± 26.7	3.5 ± 0.2	0.26	1.00
	V1	K159E/T177A	2245.3 ± 242.5	510.7 ± 12.4	4.5 ± 0.6	0.39	1.49
	V2	K77A/K159E/T177A	3092.8 ± 27.6	580.2 ± 10.5	5.3 ± 0.1	0.46	1.77
	V3	K159E/T173N/T177A	1595.7 ± 4.9	328.9 ± 21.5	4.9 ± 0.3	0.42	1.62
	V4	K159E/T177A/H254G	703.6 ± 6.9	262.7 ± 6.9	2.7 ± 0.1	0.23	0.89
	V5	K159E/T173N/T177A/H254G	763.2 ± 2.2	349.0 ± 15.9	2.2 ± 0.1	0.19	0.73
	V6	K77A/K159E/T173N/T177A/H254G	1148.0 ± 118.3	691.5 ± 64.3	1.7 ± 0.1	0.14	0.55
PNPDPP	WT		83.8 ± 2.7	51.3 ± 1.4	1.6 ± 0.01	1.00	-
	V0	(OPH-11M)	28.7 ± 0.3	28.7 ± 0.8	1.0 ± 0.02	0.61	1.00
	V1	K159E/T177A	46.8 ± 0.1	11.6 ± 0.1	4.0 ± 0.03	2.46	4.02
	V2	K77A/K159E/T177A	67.2 ± 2.2	17.5 ± 1.9	3.0 ± 0.16	1.87	3.05
	V3	K159E/T173N/T177A	48.5 ± 1.4	22.1 ± 1.9	3.5 ± 0.37	2.15	3.50
	V4	K159E/T177A/H254G	33.3 ± 0.1	18.0 ± 0.4	3.6 ± 0.14	2.23	3.65
	V5	K159E/T173N/T177A/H254G	34.5 ± 4.7	13.9 ± 2.9	2.3 ± 0.04	1.42	2.31
	V6	K77A/K159E/T173N/T177A/H254G	19.1 ± 0.1	11.0 ± 1.1	2.2 ± 0.27	1.36	2.22
DFP	WT		5008.7 ± 22.1	262.3 ± 8.1	19.1 ± 0.5	1.00	-
	V0	(OPH-11M)	562.1 ± 5.8	31.7 ± 2.8	17.8 ± 1.4	0.93	1.00
	V1	K159E/T177A	875.3 ± 1.4	33.0 ± 0.3	26.5 ± 0.3	1.39	1.49
	V2	K77A/K159E/T177A	822.1 ± 3.0	22.1 ± 0.1	37.2 ± 0.3	1.95	2.09
	V3	K159E/T173N/T177A	794.7 ± 5.8	19.9 ± 0.9	39.9 ± 1.5	2.09	2.25
	V4	K159E/T177A/H254G	4829.9 ± 674.6	461.5 ± 159.7	10.9 ± 2.3	0.57	0.61
	V5	K159E/T173N/T177A/H254G	4263.7 ± 866.5	1758.2 ± 127.8	2.8 ± 0.9	0.14	0.16
	V6	K77A/K159E/T173N/T177A/H254G	2132.7 ± 327.1	1615.5 ± 355.7	1.3 ± 0.1	0.07	0.07

^a^Substitutions relative to V0 (OPH-11M). WT, wild type; N.D., not determined.

**Table 3:** Catalytic rates of GV-hybrids.

Substrate	Clone	Mutational composition	*k*_cat_(min^-1^)	*K*_m_(μM)	*k*_cat_/*K*_m_ (×10^6^M^-1^min^-1^)	Fold increase to G0	Fold increase to V0	Fold increase to GV0

Paraoxon	G0	(rePON1-4E9)	9.1 ± 0.01	29.4 ± 0.6	0.3 ± 0.01	1.0	-	-
	V0	(OPH-11M)	705.3 ± 16.59	220.4 ± 1.1	3.2 ± 0.06	10.4	1.0	-
	GV0	G0-V0 hybrid	146.1 ± 8.26	27.7 ± 4.7	5.3 ± 0.59	17.2	1.7	1.0
	GV1	G0-V1 hybrid	3024.7 ± 99.40	214.4 ± 9.0	14.1 ± 0.13	45.7	4.4	2.7
	GV2	G9-V2 hybrid	3271.3 ± 47.17	247.9 ± 4.7	13.2 ± 0.06	42.7	4.1	2.5
	GV3	G9-V3 hybrid	2366.3 ± 35.38	219.3 ± 6.7	10.8 ± 0.49	35.0	3.4	2.0
	GV4	G10-V2 hybrid	3061.6 ± 29.20	184.9 ± 12.5	16.6 ± 0.96	53.7	5.2	3.1
	GV5	G10-V3 hybrid	1541.1 ± 25.27	105.3 ± 4.7	14.7 ± 0.42	47.5	4.6	2.8
	GV6	G11-V2 hybrid	1676.6 ± 59.53	105.3 ± 13.0	16.0 ± 1.41	51.8	5.0	3.0
	GV7	G11-V3 hybrid	3258.6 ± 51.67	54.5 ± 0.9	59.8 ± 0.06	193.7	18.7	11.3
PNPDPP	G0	(rePON1-4E9)	4.0 ± 0.2	23.0 ± 2.5	0.2 ± 0.01	1.0	-	-
	V0	(OPH-11M)	29.0 ± 2.3	31.1 ± 5.3	0.9 ± 0.09	5.4	1.0	-
	GV0	G0-V0 hybrid	17.0 ± 0.3	3.0 ± 0.1	5.7 ± 0.20	33.0	6.2	1.0
	GV1	G0-V1 hybrid	73.0 ± 1.0	16.7 ± 2.1	4.4 ± 0.48	25.0	4.7	0.8
	GV2	G9-V2 hybrid	96.3 ± 3.0	29.2 ± 0.8	3.3 ± 0.20	18.8	3.5	0.6
	GV3	G9-V3 hybrid	68.6 ± 2.2	37.4 ± 2.5	2.1 ± 0.88	12.1	2.3	0.4
	GV4	G10-V2 hybrid	81.9 ± 1.6	21.7 ± 5.4	3.8 ± 0.47	21.8	4.1	0.7
	GV5	G10-V3 hybrid	65.3 ± 4.6	16.0 ± 5.6	4.3 ± 1.22	24.4	4.6	0.8
	GV6	G11-V2 hybrid	98.0 ± 7.2	21.6 ± 3.6	4.6 ± 0.42	26.0	4.9	0.8
	GV7	G11-V3 hybrid	155.5 ± 3.7	29.7 ± 0.6	5.2 ± 0.02	29.8	5.6	0.9
DFP	G0	(rePON1-4E9)	176.8 ± 17.3	86.5 ± 30.1	2.1 ± 0.5	1.0	-	-
	V0	(OPH-11M)	556.2 ± 9.1	31.1 ± 1.9	17.9 ± 0.8	8.4	1.0	-
	GV0	G0-V0 hybrid	621.5 ± 6.8	519.5 ± 31.7	1.2 ± 0.1	0.6	0.1	1.0
	GV1	G0-V1 hybrid	882.4 ± 27.0	29.6 ± 4.6	30.1 ± 3.7	14.1	1.7	25.1
	GV2	G9-V2 hybrid	825.2 ± 13.5	22.3 ± 4.9	37.9 ± 7.8	17.8	2.1	31.6
	GV3	G9-V3 hybrid	685.6 ± 60.7	140.7 ± 42.6	5.0 ± 1.1	2.4	0.3	4.2
	GV4	G10-V2 hybrid	908.6 ± 2.2	31.5 ± 3.2	29.0 ± 3.0	13.6	1.6	24.2
	GV5	G10-V3 hybrid	899.4 ± 1.7	19.4 ± 5.9	48.7 ± 14.9	22.8	2.7	40.6
	GV6	G11-V2 hybrid	838.3 ± 32.0	35.6 ± 9.1	24.2 ± 5.3	11.3	1.4	20.2
	GV7	G11-V3 hybrid	1039.2 ± 2.8	22.3 ± 2.9	47.0 ± 6.0	22.0	2.6	39.2

Substrate	Clone	Mutational composition	*k*_cat_(min^-1^)	*K*_m_(μM)	*k*_cat_/*K*_m_ (M^-1^min^-1^)	Fold increase to G0	Fold increase to V0	Fold increase to GV0

DSM	G0	(rePON1-4E9)	0.02 ± 0.01	1054.6 ± 934.1	14.6 ± 1.9	1.0	-	-
	V0	(OPH-11M)	0.13 ± 0.01	1637.5 ± 252.4	77.5 ± 9.8	5.3	1.0	-
	GV0	G0-V0 hybrid	0.06 ± 0.09	1210.6 ± 612.9	5.0 ± 1.1	0.3	0.1	1.0
	GV1	G0-V1 hybrid	0.22 ± 0.01	748.9 ± 181.4	295.6 ± 52.9	20.3	3.8	59.1
	GV2	G9-V2 hybrid	0.17 ± 0.02	832.2 ± 236.8	214.0 ± 38.4	14.7	2.8	42.8
	GV3	G9-V3 hybrid	0.12 ± 0.01	680.1 ± 354.4	206.6 ± 90.1	14.2	2.7	41.3
	GV4	G10-V2 hybrid	0.23 ± 0.01	708.6 ± 99.5	324.0 ± 28.9	22.2	4.2	64.8
	GV5	G10-V3 hybrid	0.29 ± 0.12	10964.5 ± 5736.8	28.1 ± 5.1	1.9	0.4	5.6
	GV6	G11-V2 hybrid	0.14 ± 0.02	593.8 ± 159.8	234.8 ± 37.9	16.1	3.0	46.9
	GV7	G11-V3 hybrid	0.22 ± 0.01	432.5 ± 45.7	504.3 ± 48.5	34.6	6.5	100.9
Malathion	G0	(rePON1-4E9)	N.D.	N.D.	N.D.	-	-	-
	V0	(OPH-11M)	0.71 ± 0.04	2211.5 ± 160.5	319.4 ± 4.8	-	1.0	-
	GV0	G0-V0 hybrid	0.21 ± 0.01	994.2 ± 33.7	212.6 ± 1.1	-	0.7	1.0
	GV1	G0-V1 hybrid	1.90 ± 0.04	2736.5 ± 14.8	695.3 ± 17.5	-	2.2	3.3
	GV2	G9-V2 hybrid	5.14 ± 0.29	6395.5 ± 450.4	803.8 ± 11.0	-	2.5	3.8
	GV3	G9-V3 hybrid	3.75 ± 0.05	7980.0 ± 43.8	470.4 ± 3.7	-	1.5	2.2
	GV4	G10-V2 hybrid	2.72 ± 0.40	7642.5 ± 139.5	356.9 ± 12.8	-	1.1	1.7
	GV5	G10-V3 hybrid	12.68 ± 3.19	27460.0 ± 7891.1	464.1 ± 17.2	-	1.5	2.2
	GV6	G11-V2 hybrid	1.27 ± 0.25	3274.0 ± 924.9	393.9 ± 34.6	-	1.2	1.9
	GV7	G11-V3 hybrid	4.15 ± 0.02	3133.5 ± 95.4	1324.0± 47.5	-	4.2	6.2

N.D., not determined.
