# Supplementary material for: Engineered Recombinant PON1-OPH Fusion Hybrids: Potentially Effective Catalytic Bioscavengers against Organophosphorus Nerve Agent Analogs
Source: J Microbiol Biotechnol. 2020 Oct 30;31(1):144–53. doi: 10.4014/jmb.2006.06044 (PMC9705692; doi:10.4014/jmb.2006.06044)
Supplement: Supplementary file 1 [file jmb-31-1-144-supple.pdf]

J. Microbiol. Biotechnol.
https://doi.org/10.4014/jmb.2006.06044

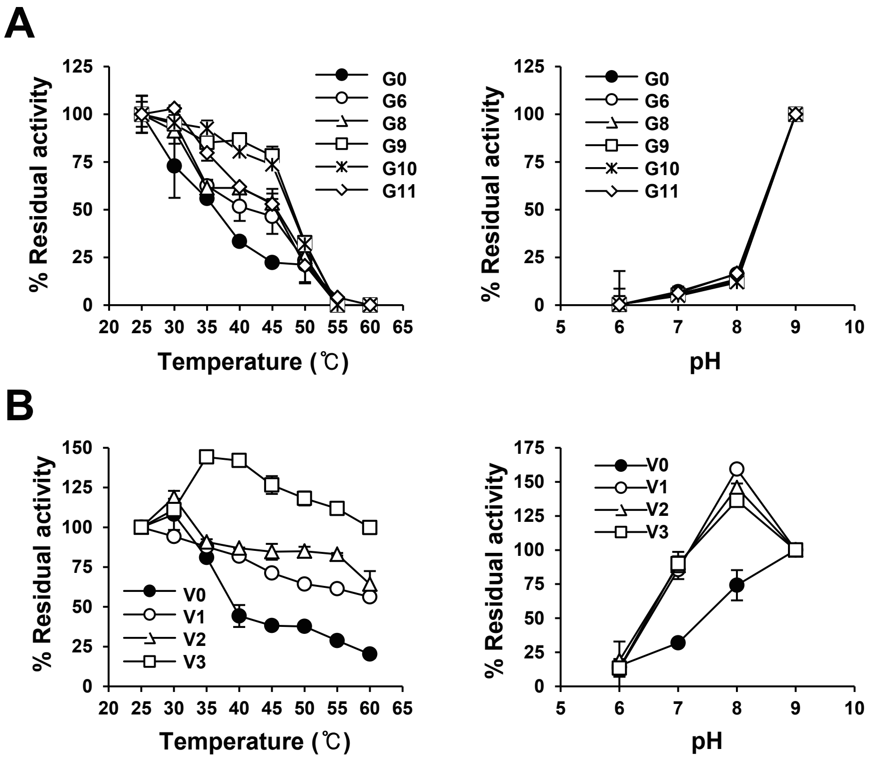

Supplementary Figure S1. Effect of temperature and pH on the purified rePON1 and OPH mutants. (A) Thermal and pH stability of purified rePON1 mutants for paraoxonase activity. Purified rePON1 mutants were preincubated at the indicated temperature (left panel) or pH (right panel) for 10 min, and then the enzymatic activity was determined with 0.6 mM paraoxon at 25 °C for 10 min. **(B)** Thermal and pH stability of purified OPH mutants for the hydrolytic activity of malathion. Purified OPH mutants were preincubated at the indicated temperature (left panel) or pH (right panel) for 10 min, and then the enzymatic activity was determined with 1.2 mM malathion at 25 °C for 10 min.

Supplementary Table S1. Catalytic activity comparison of GV-hybrids with variable linker length.

Substrate	Clone	k_{cat} (min^{-1})	K_m (μM)	k_{cat}/K_m (×10^6 M^{-1} min^{-1})	Fold increase to GV0
Paraoxon	GV0 (1×(G4S))	N.D.	N.D.	N.D.	-
	GV0	505.9 ± 25.7	91.9 ± 18.4	5.6 ± 0.84	1.0
	GV0 (3×(G4S))	11.2 ± 0.08	38.6 ± 2.4	0.3 ± 0.02	0.1
	GV7 (1×(G4S))	755.6 ± 11.01	55.2 ± 1.1	13.7 ± 0.48	2.4
	GV7	5150.3 ± 107.8	92.9 ± 3.6	55.5 ± 1.04	9.9
	GV7 (3×(G4S))	2493.3 ± 0.56	98.2 ± 2.2	25.4 ± 0.58	4.5
**Substrate**	**Clone**	**k_{cat} (min^{-1})**	**K_m (μM)**	**k_{cat}/K_m (M^{-1} min^{-1})**	**Fold increase to GV0**
Malathion	GV0 (1×(G4S))	N.D.	N.D.	N.D.	-
	GV0	0.15 ± 0.01	745.8 ± 24.0	198.7 ± 8.1	1.0
	GV0 (3×(G4S))	0.22 ± 0.01	1014.8 ± 182.8	219.2 ± 25.0	1.1
	GV7 (1×(G4S))	0.45 ± 0.04	1343.0 ± 166.9	336.5 ± 9.0	1.7
	GV7	1.71 ± 0.02	1145.5 ± 113.8	1495.7 ±129.0	7.5
	GV7 (3×(G4S))	2.29 ± 0.10	3142.0 ± 202.2	728.5 ± 16.2	3.7

The catalytic activities of GV-hybrid clones with (1×(G_4S)-linker or (3×(G_4S)-linker were compared with GV-hybrid clones with 2×(G_4S)-linker. N.D., not determined.

Supplementary Table S2. Catalytic activity comparison of GV- and VG-hybrids.

Substrate	Clone	k_{cat} (min^{-1})	K_m (μM)	k_{cat}/K_m (×10^6 M^{-1} min^{-1})	Fold increase to GV0
Paraoxon	**GV0**	379.0 ± 1.3	78.01 ± 2.7	**4.9 ± 0.18**	**1.0**
	**VG0**	6.1 ± 0.06	22.52 ± 1.0	**0.3 ± 0.01**	**0.1**
	**GV7**	4791.0 ± 71.3	89.09 ± 4.3	**53.8 ± 1.83**	**11.0**
	**VG7**	461.1 ± 2.25	56.92 ± 1.3	**8.1 ± 0.15**	**1.7**
**Substrate**	**Clone**	**k_{cat} (min^{-1})**	**K_m (μM)**	**k_{cat}/K_m (M^{-1} min^{-1})**	**Fold increase to GV0**
Malathion	**GV0**	0.15 ± 0.02	841.9 ± 73.3	**181.4 ± 12.5**	**1.0**
	**VG0**	N.D.	N.D.	**N.D.**	**-**
	**GV7**	3.7 ± 0.82	2662.0 ± 616.6	**1389.5 ± 13.4**	**7.7**
	**VG7**	0.56 ± 0.12	1432.5 ± 550.8	**408.4 ± 75.0**	**2.3**

VG-hybrid clones (OPH-(2×(G_4S))-rePON1) were constructed in reverse order of the designed GV-hybrids (rePON1-(2×(G_4S))-OPH). N.D., not determined.
